# The association between social media use and body dysmorphic symptoms in young people

**DOI:** 10.3389/fpsyg.2023.1231801

**Published:** 2023-08-17

**Authors:** Monica Gupta, Amita Jassi, Georgina Krebs

**Affiliations:** ^1^Department of Psychology, Institute of Psychiatry, Psychology & Neuroscience, King’s College London, London, United Kingdom; ^2^National and Specialist OCD and Related Disorders Clinic for Young People, South London and Maudsley NHS Foundation Trust, London, United Kingdom; ^3^Research Department of Clinical, Educational and Health Psychology, University College London, London, United Kingdom; ^4^MRC Social, Genetic and Developmental Psychiatry Centre, Institute of Psychiatry, Psychology & Neuroscience, King’s College London, London, United Kingdom

**Keywords:** social media, young people, adolescents, body dysmorphic disorder, perfectionism

## Abstract

**Introduction:**

Social media use (SMU) is highly prevalent amongst young people and previous research suggests an association with mental health problems, including poor body image. However, the potential relationship between SMU and body dysmorphic disorder (BDD) has received little attention. Furthermore, little is known about the factors that moderate the potential association between SMU and body dysmorphic symptoms. The current study tested the associations between three facets of SMU and body dysmorphic symptoms and explored perfectionism as a moderator in a non-clinical sample.

**Method:**

Two-hundred and nine 16-18-year-olds (mean age = 16.5 years, 37% male) recruited from schools in London completed an online survey measuring aspects of SMU, including: frequency of image-and text-based SMU; motivations for SMU (appearance, popularity, connection or values and interests); and active and passive SMU. Participants additionally completed validated measures of body dysmorphic symptoms, perfectionism, and anxiety/depressive symptoms. Linear regression models tested the association of body dysmorphic symptoms with different facets of SMU, with and without adjustment for age, sex and anxiety/depressive symptoms.

**Results:**

Frequency of use of image-based, but not text-based, platforms was significantly and positively associated with body dysmorphic symptoms, and this association remained significant in the adjusted models. Appearance-based motivation for SMU was the only motivator uniquely associated with body dysmorphic symptoms across the unadjusted and adjusted models. Passive, not active, SMU was associated with body dysmorphic symptoms in unadjusted models, but this association became non-significant in the adjusted models. Self-oriented perfectionism moderated the association between frequency of image-based SMU and body dysmorphic symptoms.

**Discussion:**

Image-based SMU, and appearance-based motivations for SMU, are positively associated with body dysmorphic symptoms. Self-oriented perfectionism may amplify the relationship between SMU and body dysmorphic symptoms. Our findings highlight the importance of a nuanced approach to examining SMU, and the need for further research to determine whether specific facets of SMU contribute to the development and/or maintenance of body dysmorphic symptoms.

## Introduction

Body Dysmorphic Disorder (BDD) is characterised by a persistent preoccupation with perceived flaws in physical appearance which are unobservable or appear slight to others (American Psychiatric Association, 2013). This preoccupation leads to significant impairment in daily living, reduced quality of life and strikingly high rates of suicide attempts (Phillips et al., 2005; Krebs et al., 2022). Previous research suggests that body dysmorphic symptoms (encompassing appearance preoccupation, repetitive behaviours, and impairment) are a dimensional construct and fall on a continuum (Bala et al., 2021). BDD represents one extreme end of this continuum and affects around 2% of the general population (Veale et al., 2016). A larger proportion of the population experience subthreshold body dysmorphic symptoms, which are associated with distress and impairment in their own right (Schneider et al., 2017). BDD typically emerges during adolescence (Bjornsson et al., 2013) and the aetiology of BDD is rooted in both genetic and environmental factors (Monzani et al., 2012). However, currently little is known about the specific environmental factors that contribute to the development and/or maintenance of body dysmorphic symptoms which, if left unchecked, may progress into diagnosable BDD. Social media is one environmental factor which has been proposed as a risk factor for body dysmorphic symptoms, potentially even contributing to a rise in the prevalence of BDD amongst youth (Kaur et al., 2020).

Social media use (SMU) is highly prevalent amongst young people. For example, it is estimated that 91–97% of British adolescents aged 12–17 years use social media (Ofcom, 2022). Social media platforms rely heavily on image-based content, much of which is highly curated and often enhanced. This content fuels the internalisation of unattainable beauty standards and the emphasis placed on appearance-related social evaluation which consequently increase appearance dissatisfaction (Anson et al., 2012; Laughter et al., 2023). Furthermore, social media provides a constant forum for appearance-based social comparison and impacts appearance-based self-objectification (Ramsey and Horan, 2018), both of which are key components in body image disorders such as BDD (Festinger, 1954; Veale, 2004; Neziroglu et al., 2008). Preliminary findings from a cross-sectional study of 200 17–35-year-olds showed that more frequent SMU was associated with higher body dysmorphic symptoms (Waqar et al., 2022). Another study found a specific association between use of *image*-based social media platforms with body dysmorphic symptoms amongst a mixed sample of adolescents and adults (*N* = 1,010, Alsaidan et al., 2020).

Taken together, the findings described above provide preliminary support for an overall link between SMU, particularly image-based social media, and body dysmorphic symptoms. However, these associations represent averages and there is likely to be substantial inter-individual variability. In this vein, previous research has highlighted the need to understand individual differences that influence an adolescent’s reaction to social media (Orben, 2020). One such individual difference may be motivations underpinning SMU. Young people have reported several benefits of SMU when motivated to use it to connect with others, for self-expression, for entertainment and to gain access to news and information (Reid and Weigle, 2014; Anderson and Jiang, 2018). However, appearance-motivated SMU is common and has been implicated in poorer body satisfaction and wellbeing in young people (Ryding and Kuss, 2020; Jarman et al., 2021). Amongst their sample of adolescents and adults, Alsaidan et al. (2020) found that celebrities and body health and beauty were two of the top interests when using social media, and that individuals with BDD were more likely to compare themselves with celebrities. Hence, a closer examination of the role of appearance-based motivation to use social media in the development of appearance concerns in young people is needed (Rodgers et al., 2021).

Another factor that may influence the association between SMU and body dysmorphic symptoms is the way in which individuals engage in social media, particularly the extent to which they engage in active use (for example, posting one’s own content) vs. passive use (for example, browsing others’ content). Whilst active SMU has been linked to some positive outcomes like increased perception of friends’ support (Frison and Eggermont, 2020), both active and passive SMU have been associated with poorer body image due to increased social comparison and exposure to negativity from peers (Rousseau et al., 2017; Hogue and Mills, 2019). It is plausible that passive SMU may be used to gather information on how to improve one’s appearance, increasing body dissatisfaction (Ryding and Kuss, 2020). Passively viewing images of ideal and fit bodies on social media, compared to viewing travel-themed images, increased body dissatisfaction and negative mood in women (Tiggemann and Zaccardo, 2015). Meanwhile, active SMU, including sharing photos of oneself and “liking” or commenting on others’ photos has been associated with an individual’s self-worth being more appearance based and with body dissatisfaction (McLean et al., 2015; Holland and Tiggemann, 2016). Whilst Jarman et al. (2021) did not find an association between SMU to seek appearance feedback and active or passive use, these variables may individually be associated with body dysmorphic symptoms.

A third factor that may moderate the association between SMU and body dysmorphic symptoms is perfectionism. Perfectionism includes having excessively high standards for oneself (self-oriented perfectionism) and beliefs that others hold one to excessively high standards (socially-prescribed perfectionism) (Hewitt and Flett, 1991). The seemingly “perfect” images displayed by peers and celebrities on social media may impact how adolescents think they need to portray themselves on these online platforms. Cognitive behavioural models of BDD propose that perfectionism is a risk factor for the development of BDD (Veale, 2004; Fang and Wilhelm, 2015; Krebs et al., 2019). In support of this, existing research has shown that socially-prescribed perfectionism is associated with body dysmorphic symptoms amongst university students (Hanstock and O'Mahony, 2002; Bartsch, 2007) and that self-oriented perfectionism predicts body dysmorphic symptoms over time (Krebs et al., 2019). Additionally, newer findings indicate that perfectionism mediates the association between social media pressure felt by young adult women and body dysmorphic symptoms (Sulistyo et al., 2022). It is therefore possible that perfectionism also plays a moderating role in the association between SMU and body dysmorphic symptoms, particularly when exposed to image-based content. This interaction is yet to be explored in young people.

In summary, there is a paucity of literature exploring the relationship between SMU and body dysmorphic symptoms. The few existing studies highlight the importance of examining the nuances of SMU in relation to body dysmorphic symptoms, including the type of social media (image-vs. text-based), motivations for SMU, and whether SMU is active or passive. Research has also highlighted the need to consider individual differences that might moderate the association of SMU with body dysmorphic symptoms.

The current study therefore aimed to expand upon the existing literature by examining the associations between three facets of SMU (frequency of image-based platforms, motivation, and active or passive) and body dysmorphic symptoms in a non-clinical sample of young people. In addition, this study aimed to explore the moderating role of perfectionism in the association between frequency of use and body dysmorphic symptoms. Based on previous empirical findings in the fields of body image and BDD, we hypothesised that higher frequency of use of image-based, but not text-based, social media platforms would be associated with body dysmorphic symptoms in young people. Second, we expected that appearance-based motivation to use social media would be more strongly associated with body dysmorphic symptoms than other motivations. Third, we predicted that there would be a positive association between each passive and active SMU and body dysmorphic symptoms. Lastly, we hypothesised that both self-oriented and socially-prescribed perfectionism would moderate the association between frequency of image-based SMU and body dysmorphic symptoms.

## Materials and methods

### Participants

This study utilised a cross-sectional, survey-based design. Several schools in South London, United Kingdom were approached with information about the study and were invited to take part via phone and email. Three schools responded and were considered appropriate as they were all government funded, co-educational and represented three different London boroughs with varied demographic makeup. Participants were recruited from Years 12 and 13 at each school. Based on correlation analysis, to detect an effect size of 0.23 with 80% power and an alpha level of 0.025, the study required 176 participants. This figure was rounded to 190 to pre-emptively account for any withdrawals or excluded data (G*Power 3.1, Faul et al., 2009). Approximately 869 young people aged 16–18 years old were invited to take part, of which 209 young people participated (64% female and 36% male), giving a response rate of approximately 24%.

### Procedure

Students were presented with verbal and written descriptions of the study and subsequently given access to a web link for the online survey, which was hosted on the digital platform Qualtrics. During the verbal presentation of the study, participants were advised to complete the surveys privately, without sharing their answers with others to prevent influencing one another. To encourage this, participants completed the survey outside of lesson time but were allowed to use school computers. Participants provided informed written consent at the start of the survey and were told that they could withdraw their data up to 1 week after submitting their responses. To ensure voluntary participation, teachers did not know which students had completed the survey, and so all students were given up to two verbal or written reminders about the study by a teacher. At the end of the survey participants could choose to enter a prize draw for a shopping voucher. Ethical approval for the study was granted by the Psychiatry, Nursing and Midwifery Research Ethics Subcommittee of King’s College London (HR/DP-20/21-2139).

### Measures

Core demographic information (age, sex, gender, ethnicity, and school year group) were collected as part of the survey along with the following measures.

The **Body Image Questionnaire-Child and adolescent version** (BIQ-C; Veale, 2009) is a 14 item self-report measure that captures the degree of impairment in the young person’s life caused by body dysmorphic symptoms. Participants were advised that responses on this measure should not focus on shape or weight concerns, but strictly the appearance of body features. The BIQ-C is based on, and is almost identical to, the Cosmetic Procedure Screening questionnaire (COPS) which has good test-retest reliability and convergent validity in adults (Veale et al., 2012). When evaluated in 12–18-year-olds, the BIQ-C showed good internal reliability (Cronbach’s α = 0.88) and ability to discriminate between adolescents with probable BDD vs. no BDD (Schneider et al., 2017). However, whilst cut-offs have been noted for the 9-item BIQ-C (Schneider et al., 2018), no cut-offs for the full BIQ-C have been set. The cut-off for the BIQ (adult version) is 59 and so this was used to indicate probable BDD in the current sample, in line with other studies in adolescents (Krebs et al., 2019). In the current study the BIQ-C showed good internal consistency (Cronbach’s α = 0.88).

Participants were asked how long they had had at least one social media account for and which accounts they had. To assess **frequency** of SMU, participants were then asked to indicate on an eight-point scale, from 0 “almost no time” to 7 “≥6 h,” how long they spent on an average weekday during term-time on image-based social media (e.g., Snapchat) and text-based social media (e.g., Twitter), and mixed social media (e.g., Facebook).

The **Multidimensional Scale of Facebook Use** (Frison and Eggermont, 2016; adapted by Thorisdottir, 2020 to include all types of social media), is a six-item questionnaire assesses how much young people use social media actively (for example posting their own content or liking and commenting on others’ content) and/or passively (for example browsing others’ content). Both subscales had acceptable internal consistency (active SMU: Cronbach’s α = 0.80, passive SMU: Cronbach’s α = 0.74; Thorisdottir, 2020). In the current study both subscales showed moderate internal consistency (active SMU: Cronbach’s α = 0.62, passive SMU: Cronbach’s α = 0.62).

The **Motivations for Social Media Use Scale** (MSMU; Rodgers et al., 2021), is a 15-item questionnaire assesses young people’s motivations for using social media. It consists of four subscales (appearance, popularity, connection and values and interests). The MSMU has recently been validated in a large sample of adolescents (*n* = 770; Cronbach’s alpha >0.78 on all subscales in boys and girls; Rodgers et al., 2021). In the current study the MSMU had good internal consistency (Cronbach’s α = 0.89).

The **Revised Child Anxiety and Depression Scale** (RCADS-11 child version; Radez et al., 2020), is a13-item scale, including 11 symptom-focused questions and two impact questions, is a measure of current symptoms of anxiety and depression in youth. It is a shortened version of the original 47-item measure, which was developed by Chorpita et al. (2000). This shortened version of the RCADS showed good internal consistency (McDonald’s omega ranged from 0.87 to 0.94) and favourable convergent validity (Radez et al., 2020). In the current study the RCADS had excellent internal consistency (Cronbach’s α = 0.93).

The **Short-Form of the Child-Adolescent Perfectionism Scale** (CAPS-SF; Bento et al., 2020), is a widely-used scale has 9-items which are rated from 1 (false, not true at all) to 5 (very true of me). The CAPS-SF comprises two subscales measuring socially-prescribed perfectionism and self-oriented perfectionism. In a sample of 9–18-year-olds, both subscales show adequate internal consistency (Cronbach’s *a* = 0.84–0.86), test-retest reliability (0.67 and 0.61, *p* = <0.001) and structural validity (CFI = 0.975, RMSEA 0.047; Bento et al., 2020). In the current study both the self-oriented and socially-prescribed subscales had good internal consistency (Cronbach’s α = 0.84 and 0.88 respectively).

### Statistical analyses

Data met assumptions of normality and multi-collinearity. The distributions of scores on the continuous measures were examined using skewness and kurtosis and all measures were normally distributed. Regarding the frequency of SMU, references to “frequency of any SMU” hereafter encompass all platforms (that is, text-based, image-based and mixed-media) whereas references to “image-based SMU,” such as in our first hypothesis, are inclusive of imaged-based and mixed-media platforms as both use imagery.

A series of multiple linear regressions were used to test the cross-sectional associations between different facets of SMU (i.e., frequency, active or passive and motivation) and body dysmorphic symptoms. Each regression was run three times, (i) without adjustments for covariates, (ii) with adjustment for age and sex, and (iii) with adjustment for age, sex and RCADS-11 score (anxiety and depression symptoms). Since symptoms of anxiety (e.g., worry) and depression (e.g., low self-esteem) are core feature of BDD, adjusting for them could be overly conservative and mask a true effect between SMU and body dysmorphic symptoms. For this reason, we intended our main interpretation to be of regression models that did not adjust for RCADS-11 score. However, we also ran the adjusted models in order to determine whether aspects of SMU were associated with aspects of BDD phenomenology independent of anxiety and depression. Moderation effects were tested using interaction terms in the regression models (i.e., interaction between passive SMU and self-orientated perfectionism and interaction between active SMU and socially-prescribed perfectionism). All analyses were completed using IBM SPSS Statistics 28.

## Results

### Sample characteristics

In total 209 young people completed the survey. One participant was excluded due to contradictory and therefore invalid responses in relation to social media use. Therefore, the final sample consisted of 208 participants.

Participant characteristics are shown in Table 1. The majority reported their natal sex as female (64%, *n* = 133). Nine participants who reported their natal sex as female reported their gender as male, non-binary/other or preferred not to say. All young people who reported their natal sex as male (36%, *n* = 75), reported their gender as male. Over two thirds of the sample were in Year 12 (68%, *n* = 141) with the majority being aged 16 (61%, *n* = 128). Regarding ethnicity, 52% of the sample identified as White British (*n* = 109).

**Table 1:** Sample characteristics (*N* = 208).

Demographic characteristics		*n*	%
Sex	Female	133	64%
	Male	75	36%
Gender	Female	124	59%
	Male	76	37%
	Non-binary/other	6	3%
	Prefer not to say	2	1%
Age	16	128	61%
	17	73	35%
	18	8	4%
School year group	12	141	68%
	13	67	32%
Ethnicity	White British	109	52%
	White Other	29	14%
	Asian	15	7%
	African or Caribbean	25	12%
	Mixed or Multiple ethnicities	22	11%
	Other	8	4%
**Clinical characteristics**		**Mean (sd)**	**Range (scale range)**
BIQ-C total score		29.02 (16.69)	0–81 (0–96)
Frequency of any SMU (hourly scale points)		6.02 (3.14)	0–17 (0–21)
Frequency of text-based SMU (hourly scale points)		1.08 (1.18)	0–7 (0–7)
Frequency of imaged-based SMU (hourly scale points)		4.95 (2.64)	0–13 (0–14)
Active SMU		10.40 (3.81)	0–18 (0–18)
Passive SMU		6.02 (3.50)	0–16 (0–18)
Motivations for SMU: Appearance		9.07 (3.92)	5–25 (5–25)
Motivations for SMU: Connections		7.88 (2.73)	3–15 (3–15)
Motivations for SMU: Values and Interests		8.28 (3.03)	3–15 (3–15)
Motivations for SMU: Popularity		7.79 (3.49)	4–17 (4–20)
CAPS: self-oriented perfectionism		14.28 (3.74)	4–20 (4–20)
CAPS: socially-prescribed perfectionism		13.84 (5.20)	5–25 (5–25)
CAPS total score		28.12 (7.26)	9–45 (9–45)
RCADS anxiety		6.94 (4.32)	0–16 (0–18)
RCADS depression		6.58 (3.94)	0–15 (0–15)
RCADS total score		13.95 (8.17)	0–33 (0–33)
RCADS total score + impact supplement		16.52 (9.59)	0–39 (0–39)

BIQ-C, body image questionnaire-child; SMU, social media use; CAPS, child and adolescent perfectionism scale; RCADS, revised child anxiety and depression scale.

The mean scores for body dysmorphic symptoms (BIQ-C), the measures of SMU, perfectionism (CAPS) and anxiety and depression (RCADS-11) are presented in Table 1. In total, nine participants (4.3%) scored above the clinical cut-off for BDD (≥59) on the BIQ-C. Participants reported a wide variety of concerning features, with acne/acne scarring (typically on the face), stomach (being “too big” or not being “flat enough”), and the nose (being too big, or crooked in some way) being the most commonly reported features of concern (see Supplementary Figure S1).

Regarding SMU, all respondents reported they had a least one social media account and on average they had an account for 5 years. The most common social media account was Instagram (*n* = 197, 94.7%), closely followed by Snapchat (*n* = 173, 83.2%) and TikTok (*n* = 150, 72.1%). Least common were Pinterest (*n* = 121, 58.2%), Twitter (*n* = 99, 47.6%) and Facebook (*n* = 47, 22.6%). Several other social media platforms were identified by a small proportion of the sample (see Supplementary Table S1).

Participants had a mean score of 6.02 for frequency of any SMU, corresponding to 5–6 h per day (Table 1). Around 4–5 h of this time is spent on image-based SMU and less than 1 h is spent on text-based SMU. Participants scored higher on active SMU than passive SMU (Table 1). Participants also scored higher on appearance-based motivation for SMU than any other motivation (Table 1).

### Cross-sectional analyses

#### The association between frequency of image-based SMU and body dysmorphic symptoms

A linear regression model showed that frequency of any SMU (including text-based, image-based and mixed-media) was significantly associated with body dysmorphic symptoms (Table 2). This remained significant when controlling for age and sex, but became non-significant when additionally adjusting for anxiety and depression (Table 2).

**Table 2:** Results of multiple linear regression models testing the association between frequency of SMU and body dysmorphic symptoms (*N* = 208).

	Without adjustment for additional variables			With adjustment for age and sex			With adjustment for age, sex and anxiety and depression		
	*t*	β*	R^2^	*t*	β*	R^2^	*t*	β*	R^2^
**Model 1**									
Frequency of any SMU	3.96***	0.266	0.071	3.07**	0.198	1.64	0.475	0.087	0.187
**Model 2**									
Frequency of image-based SMU	3.96***	0.274	0.078	3.371***	0.223	0.195	1.801	0.099	0.476
Frequency of text-based SMU	0.272	0.019		−0.269	−0.018		−0.138	−0.007	

Outcome variable: BIQ-C total score.

SMU, social media use; β*, Standardised Beta coefficient; **p < 0.01, ***p < 0.001.

These analyses were repeated for image-based SMU and text-based SMU separately. Frequency of text-based SMU was not significantly associated with body dysmorphic symptoms (Table 2). In contrast, frequency of image-based SMU (including purely image-based, such as Snapchat, and mixed-media, such as Instagram) was significantly associated with body dysmorphic symptoms. This association remained significant when controlling for age and sex with a 0.22 increase in body dysmorphic symptoms for every hour increase in frequency of SMU (Table 2). The association was no longer significant when additionally controlling for anxiety and depression symptoms.

#### The association between motivation to use social media and body dysmorphic symptoms

A multivariable linear regression showed that appearance-based motivation for SMU was the only type of motivation that was uniquely and positively associated with body dysmorphic symptoms (Table 3). This association remained significant when controlling for age and sex and when also adjusting for anxiety and depression symptoms.

**Table 3:** Results of a multivariate regression analysis testing the association between motivations for SMU and body dysmorphic symptoms (*N* = 208).

	Without adjustment for demographic or clinical variables		With adjustment for age and sex			With adjustment for age, sex and anxiety and depression			
	*t*	β*	R^2^	*t*	β*	R^2^	*t*	β*	R^2^
**Model 1**									
Appearance	3.347***	0.309	0.165	2.260*	0.210	0.225	1.1988*	0.149	0.502
Connection	1.117	0.087		0.416	0.032		0.364	0.022	
Values	1.417	0.097		0.657	0.045		−0.060	−0.003	
Popularity	0.060	0.006		0.723	0.069		0.702	0.054	

Outcome variable: BIQ-C total score.

The four motivation subscales were entered into each model together; β*, Standardised Beta coefficient; *p < 0.05, ***p < 0.001.

#### The association of passive and active SMU with body dysmorphic symptoms

A multivariable linear regression indicated that passive SMU, but not active SMU, was significantly, uniquely associated with body dysmorphic symptoms (Table 4). However, when additionally controlling for age and sex the association of passive SMU with body dysmorphic symptoms was no longer significant but conversely, active SMU emerged as significant (Table 4).

**Table 4:** Results of a multivariate regression analysis testing the association between active and passive SMU and body dysmorphic symptoms (*N* = 208).

	Without adjustment for demographic or clinical variables			With adjustment for age and sex			With adjustment for age, sex and anxiety and depression		
	*t*	β*	R^2^	*t*	β*	R^2^	*t*	β*	R^2^
**Model 1**									
Passive SMU	2.11*	0.151	0.049	1.496	0.102	0.170	0.871	0.047	0.488
Active SMU	1.71	0.122		1.179	0.080		2.361*	0.126	

Outcome variable: BIQ-C total score.

SMU, social media use; β*, Standardised Beta coefficient; *p < 0.05.

#### The role of perfectionism in the association between SMU and body dysmorphic symptoms

A multivariable linear regression, which included the interaction term frequency of image-based SMU x self-oriented perfectionism, was used to explore how far self-oriented perfectionism moderates the association between image-based SMU and body dysmorphic symptoms. A significant interaction effect was found which suggests that self-oriented perfectionism moderated the association between frequency of image-based SMU and body dysmorphic symptoms (Table 5). This remained true when controlling for age, sex and RCADS-11 score.

**Table 5:** Results of multiple linear regression models testing the interaction effect between frequency of use of imaged-based social media and perfectionism on body dysmorphic symptoms (*N* = 208).

	With adjustment for the main effect variables			With adjustment for the main effect variables age and sex			With adjustment for the main effect variables age, sex, anxiety and depression		
	*t*	β*	R^2^	*t*	β*	R^2^	*t*	β*	R^2^
**Model 1**									
Frequency of image-based SMU × self-oriented perfectionism	3.516***	1.074	0.183	3.122**	0.922	0.255	2.458*	0.600	0.502
**Model 2**									
Frequency of image-based SMU × socially-prescribed perfectionism	0.166	−0.064	−0.284	0.258	−0.076	−0.352	0.489	−0.150	−0.837

Outcome variable: BIQ-C total score.

SMU, social media use; β*, Standardised Beta coefficient; **p < 0.01, ***p < 0.001.

A second multivariable linear regression was run, which included the interaction term frequency of image-based SMU x socially-prescribed perfectionism. There was no significant interaction effect between active SMU and socially-prescribed perfectionism (Table 5). That is, socially-prescribed perfectionism did not moderate the association between image-based SMU and body dysmorphic symptoms. This remained the case when controlling for age, sex and RCADS-11 score.

## Discussion

Given the high prevalence of both SMU and BDD in young people, the current cross-sectional study sought to contribute to the scarce but growing field of research exploring the association between young people’s SMU and body dysmorphic symptoms. In our sample of 208 16-to 18-year-olds, greater frequency of SMU was associated with higher self-reported body dysmorphic symptoms. Of note, this association was specific to social media platforms which are highly image-based (such as Instagram and TikTok), as opposed to text-based platforms (such as Twitter). This finding is in line with existing literature (Alsaidan et al., 2020; Jarman et al., 2021). Moreover, although the direction of effect cannot be inferred in the current study, this finding is consistent with the notion that social media may play a role in the development or maintenance of body dysmorphic symptoms.

In addition, the current study found that young people with higher appearance-motivated SMU also had more self-reported body dysmorphic symptoms, whereas other motivations for SMU were not uniquely associated with body dysmorphic symptoms. This is in line with research which found that individuals who had more appearance concerns had more emotional attachment to social media sites and they would use social media in an appearance-motivated manner (Rutledge et al., 2013). Qualitative research highlights how adults with diagnosed BDD report being aesthetically motivated and feel a duty to look good to increase positive judgements from others and success in life (Silver et al., 2010), values which are likely to be played out online. Appearance-motivated SMU includes making appearance-based comparisons when online, which is known to be more common in people with BDD (Alsaidan et al., 2020) and which mediates the relationship between SMU and body image dissatisfaction (Ryding and Kuss, 2020).

In the current study, passive SMU (looking at others’ content but not posting content) was significantly associated with body dysmorphic symptoms, although this relationship became non-significant when adjusting for age and sex. This may imply that *how* an individual engages with social media is of less relevance with regards to their body dysmorphic symptoms than their motivations for SMU, the type of material they are exposed to (namely image-based material) and for how long. Interestingly, the association between *active* SMU (e.g., posting content) and body dysmorphic symptoms became marginally significant (standardised beta = 0.13; *p* = 0.019) when controlling for anxiety and depressive symptoms. This finding is unexpected, and possibly spurious. However, it is possible that active SMU is related to non-anxiety and non-depressive aspects of BDD phenomenology, such as appearance-focused repetitive behaviours. Existing research has postulated that taking selfies is a safety behaviour which perpetuates body dysmorphic symptoms (Khanna and Sharma, 2017). Posting these selfies online could constitute active SMU and therefore it is possible that selfie taking is a mediator in the association between active SMU and body dysmorphic symptoms. Although the retrospective self-report of active and passive SMU may be imprecise (Ryding and Kuss, 2020) these findings may offer the first insight into if and how passive and active SMU may relate to body dysmorphic symptoms.

Finally, we found that the association between frequency of image-based SMU and self-reported body dysmorphic symptoms was moderated by self-oriented perfectionism. That is, the relationship between image-based SMU and body dysmorphic symptoms was stronger amongst individuals with higher levels of self-oriented perfectionism. This finding is consistent with previous research showing that that self-oriented, but not socially-prescribed, perfectionism is a risk factor for body dysmorphic symptoms (Krebs et al., 2019). Furthermore, discrepancy perfectionism, occurring when there is excessive focus on the discrepancy between one’s standards for oneself and one’s actual performance (Slaney et al., 2001), has been found to mediate the path between social media pressure and body dysmorphic symptoms (Sulistyo et al., 2022). Taken together, the findings of these studies raise the possibility that individuals with higher levels of self-oriented perfectionism are more likely to compare themselves with idealised images of beauty and to become self-critical if they perceive their own appearance as falling short of these standards, which in turns fuels body dysmorphic symptoms. Interestingly, in the current study socially-prescribed perfectionism did not moderate the association between frequency of image-based SMU and body dysmorphic symptoms, which is in keeping with previous research suggesting that individuals with BDD place higher value on meeting their own aesthetic standards than on meeting others’ aesthetic expectations of them (Krebs et al., 2019).

Overall, the findings from this study imply that SMU and body dysmorphic symptoms are linked. Though the direction of effect has not been explored, it is plausible that a bidirectional relationship exists between these variables. That is, more frequent appearance-based and appearance-motivated SMU increases exposure to unattainable appearance ideals and consequently negatively impacts body dysmorphic symptoms. Meanwhile, those with higher body dysmorphic symptoms, and higher self-oriented perfectionism, are more likely to engage in unhelpful appearance-related SMU, for example making upward social comparisons and seeking reassurance about their appearance. Thus, a self-perpetuating cycle is formed in which both body dysmorphic symptoms and SMU become more ingrained. Our findings indicate that when working clinically with young people with body dysmorphic symptoms, conversations should go beyond asking about the frequency of SMU, and additionally explore the type of platforms young people are using most as well as their motivations for SMU. Such exploration may highlight when excessive appearance-related SMU may be interacting negatively with a young person’s body dysmorphic symptoms and protective measures can be identified, such as limiting time spent on image-based platforms or finding value in alternative reasons for using social media that are not appearance-focused.

### Limitations

The first limitation relates to the generalisability of the sample. Whilst participants were from wide ranging ethnic backgrounds, the sample was skewed towards females living in an urban setting, the majority of whom were 16 years old, and therefore it cannot be assumed that findings would generalise to other demographic groups. Additionally, the voluntary participation may have introduced response bias with those interested in body image, social media and psychology research being more likely to participate. Furthermore, there was a relatively low response rate to the survey, which may be partly attributed to the fact that students did not have an allotted time to complete the survey during school hours.

A second limitation of this study is the recruitment of a non-clinical sample, which may limit the inferences that can be drawn about an association between SMU and diagnosable BDD. However, recent research has demonstrated that body dysmorphic symptoms are likely to be a dimensional construct with BDD representing one extreme end of a continuum rather than being qualitatively distinct from individuals without. Thus, data collected from the general population is likely to capture the full continuum of body dysmorphic symptoms, including individuals at the extreme end who would qualify for a diagnosis of BDD if properly assessed. A dimensional approach has the advantage of maximising variance and therefore statistical power. Furthermore, since BDD is a strikingly underdiagnosed disorder, recruiting individuals with a diagnosis of BDD, such as those attending specialist clinics, may result in a biased sample.

The definition and measurement of body dysmorphic symptoms could be considered a third limitation of this study. Reliance on self-report assessment of body dysmorphic symptoms may be problematic, given that individuals with BDD typically have impaired insight into their symptoms (Phillips et al., 2012). Furthermore, in the absence of a clinical assessment, it is not possible to determine whether preoccupations relate to ‘perceived’ flaws or objective, visible differences in appearance. Additionally, self-report measures of BDD may also capture other, non-BDD, body dissatisfaction symptoms such as eating disorder psychopathology. However, in the current study we attempted to minimise the likelihood of capturing non-BDD psychopathology by explicitly stating in the instructions of the BDD measure that participants should only report appearance concerns that did not relate to shape or weight. Furthermore, attempts to capture BDD-related psychopathology were made by using a validated measure of body dysmorphic symptoms (the BIQ-C), which assesses appearance preoccupation as well as repetitive behaviours, distress, and impairment. These factors map directly onto diagnostic criteria for BDD and differentiate BDD from general body dissatisfaction (American Psychiatric Association, 2013). Equally, our definition of ‘body dysmorphic symptoms’ mirrored the diagnostic criteria for BDD, to increase clarity regarding the symptoms measured and discussed in this study. However, it should be noted that there is no consensus in the literature on how the term ‘body dysmorphic symptoms’ should be defined, and in some studies it may encompass high levels of general body dissatisfaction. The results of the current study indicated that prevalence of young people who scored above the clinical cut-off for BDD was in line with population prevalence estimates (Veale et al., 2016; Krebs et al., 2019) as were the most commonly reported features of concern; skin, face and stomach (Phillips et al., 2006; Alsaidan et al., 2020). Though a BDD diagnosis could not be confirmed without a clinician-administered assessment, these findings provide some indirect support for the validity of the measure used.

Finally, the fourth limitation to consider was this study’s reliance on cross-sectional data, from which directions of effect cannot be inferred. Future research could employ an experimental or longitudinal design in order to explore the causal relationship between SMU and body dysmorphic symptoms.

## Conclusion

In summary, this is one of the first studies to link SMU with body dysmorphic symptoms in young people, in line with literature that has already associated SMU with a higher symptom severity of other mental health difficulties (Kelly et al., 2018). Our findings go further, showing that more frequent use of image-based social media platforms and appearance-based motivation for SMU have a particular role to play in body dysmorphic symptoms, which is consistent with the aesthetic focus of clinical BDD. Novel evidence has also emerged demonstrating that self-oriented perfectionism moderates the association between SMU and body dysmorphic symptoms in young people. Our findings highlight the importance of separating the components of perfectionism when examining its role further. A causal relationship between these variables should be explored and further research is essential to understand how to minimise social media-related risks which may instigate new or maintain existing body dysmorphic symptoms.

## Data availability statement

Aggregate data may be made available on request. Requests to access the data can be made the corresponding author.

## Ethics statement

This study involving humans was approved by the PNM Research Ethics Subcommittee, King’s College London. The study was conducted in accordance with the local legislation and institutional requirements. The participants provided their written informed consent to participate in this study.

## Author contributions

This study was conducted as part of MG’s doctoral thesis, under the supervision of GK and AJ. MG, AJ, and GK contributed to conception and design of the study. MG collected the data, managed the database, performed the statistical analysis, and wrote the drafts of the manuscript. GK revised every draft of the manuscript. All authors contributed to the article and approved the submitted version.

## Funding

During the period of this research, Georgina Krebs was funded by an MRC Clinical Research Training Fellowship (MR/N001400/1).

## Conflict of interest

The authors declare that the research was conducted in the absence of any commercial or financial relationships that could be construed as a potential conflict of interest.

## Publisher’s note

All claims expressed in this article are solely those of the authors and do not necessarily represent those of their affiliated organizations, or those of the publisher, the editors and the reviewers. Any product that may be evaluated in this article, or claim that may be made by its manufacturer, is not guaranteed or endorsed by the publisher.
