# Supplementary material for: The association between social media use and body dysmorphic symptoms in young people
Source: Front Psychol. 2023 Aug 17;14:1231801. doi: 10.3389/fpsyg.2023.1231801 (PMC10471190; doi:10.3389/fpsyg.2023.1231801)
Supplement: Supplementary file 1 [file Data_Sheet_1.docx]

Supplementary Material

**The association between social media use and body dysmorphic symptoms in young people**

**Monica Gupta^1,2^, Amita Jassi^2^, Georgina Krebs^2,3,4^***

1. Department of Psychology, Institute of Psychiatry, Psychology & Neuroscience, King’s College London, London, UK
2. National and Specialist OCD and Related Disorders Clinic for Young People, South London and Maudsley NHS Foundation Trust, London, UK
3. Research Department of Clinical, Educational and Health Psychology, University College London, UK
4. MRC Social, Genetic and Developmental Psychiatry Centre, Institute of Psychiatry, Psychology & Neuroscience, King's College London, London, UK

***Correspondence:**

Dr Georgina Krebs

g.krebs@ucl.ac.uk

**Figure S1. Pie chart showing the proportion of the top two physical features of concern reported by young people**


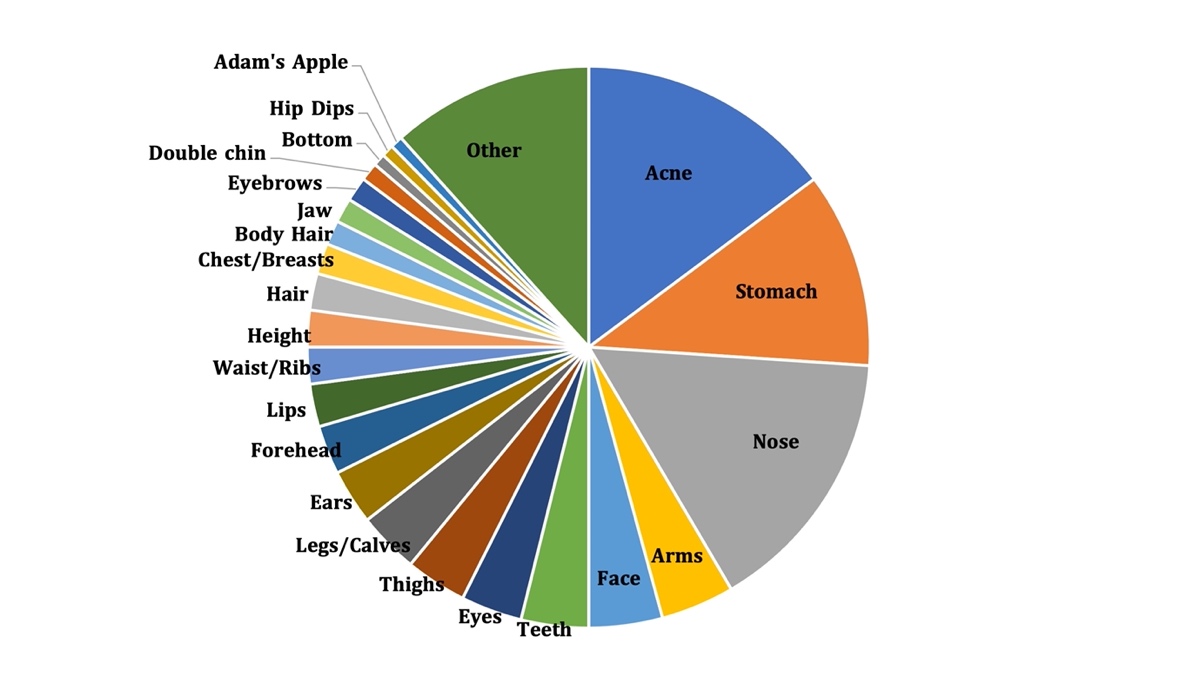


| **Table S1. Frequency of young people using specific social media platforms** | | |
| --- | --- | --- |
| Social Media Platform | Frequency (*n*) | Percentage of sample (%) |
| Instagram | 197 | 94.7 |
| Snapchat | 173 | 83.2 |
| TikTok | 150 | 72.1 |
| Pinterest | 121 | 58.2 |
| Twitter | 99 | 47.6 |
| Facebook | 47 | 22.6 |
| Other  Discord  YouTube  Reddit  WhatsApp | 17  15  10  8 | 8.2  7.2  4.8  3.8 |
| *Note.* All ‘other’ platforms were reported in open text | | |

**
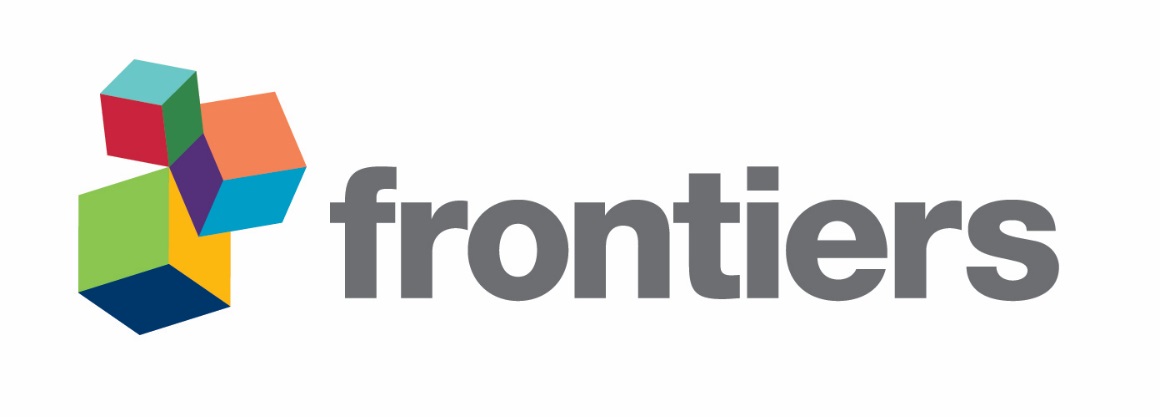
**
